# Statistical, Morphometric, Anatomical Shape Model (Atlas) of Calcaneus

**DOI:** 10.1371/journal.pone.0134603

**Published:** 2015-08-13

**Authors:** Aleksandra U. Melinska, Patryk Romaszkiewicz, Justyna Wagel, Marek Sasiadek, D. Robert Iskander

**Affiliations:** 1 Department of Biomedical Engineering, Wroclaw University of Technology, Wroclaw, Poland; 2 Regional Specialist Hospital, Research and Development Centre, Wroclaw, Poland; 3 Department of General Radiology, Interventional Radiology and Neuroradiology, Wroclaw Medical University, Wroclaw, Poland; Chinese Academy of Sciences, CHINA

## Abstract

The aim was to develop a morphometric and anatomically accurate atlas (statistical shape model) of calcaneus. The model is based on 18 left foot and 18 right foot computed tomography studies of 28 male individuals aged from 17 to 62 years, with no known foot pathology. A procedure for automatic atlas included extraction and identification of common features, averaging feature position, obtaining mean geometry, mathematical shape description and variability analysis. Expert manual assistance was included for the model to fulfil the accuracy sought by medical professionals. The proposed for the first time statistical shape model of the calcaneus could be of value in many orthopaedic applications including providing support in diagnosing pathological lesions, pre-operative planning, classification and treatment of calcaneus fractures as well as for the development of future implant procedures.

## Introduction

Improving the diagnosis and supporting the therapy is a fundamental task of medical imaging [1, 2]. Planning the surgery and choosing the method of surgical treatment often requires deep knowledge of the shape and morphological characteristics of an object at hand. In case of traumatology the detailed assessment of the morphological characteristics of bone is important for the choice of stabilization method and subsequent healing process. Approximately 10% of all fractures occur in foot bones [3]. The calcaneus (*lat*.os calcis) fracture is the most common fracture of tarsal bones, with an incidence of about 60% [4, 5]. Calcaneus fractures are estimated at 2% of all fractures of human bones and can have long-term consequences for patients in terms of comfort and disability [6, 7]. This largest tarsal bone plays stabilizing function for human body and pathologies of this bone influence human mobility and quality of life [8–10]. The evaluation of calcaneus fractures and determination of the effect of treatment essentially uses three types of imaging techniques: X-ray CT, and MRI [11–15]. Although X-ray study (radiography) remains the method of choice for the initial assessment of fracture [16, 17], CT imaging has become the current state-of-art in diagnostics of calcaneus fractures [18–20]. An invaluable advantage of CT is the possibility of reconstructing the scanned object. The location and configuration at a joint can be provided having 3D morphological and architectural information about the individual foot bones [21]. Several studies considered characterizing the calcaneal bone shape. Early works of Stindel et at. [21, 22] focused on classification of foot type in terms of pathological deformations using MRI. Stephan et al. [23] obtained information on the joint surfaces and 3D orientations of calcaneus using CT to quantify the integrity of calcaneus joint surfaces. Guterkunst et al. [24] aimed at assessing the measurement precision of landmark-based 3D bone-to-bone orientations of hind foot and lesser tarsal bones to compare an atlas-based automated method to the expert rating. Qiang et al. [7] described morphological characteristics of the calcaneus based on CT images. The survey in the later the paper has an epidemiological value. They described calcaneus by size and distances between characteristic points on the surface that were manually outlined by an experienced operator. Hence, there are possibilities of bone shape description from which a model of calcaneus could be constructed. This subsequently can be used for building statistical anatomical atlases (statistical shape models, SSMs) [25, 26]. Many tasks in medical imaging involve automatic systems which use prior knowledge about an object. Model based methods have become popular because they have the potential to resolve possible confusion associated with structural complexity, to improve tolerance for noisy data and deliver more accurate results in case of missing data [27–32]. In medical imaging, SSMs are mainly used in segmentation and recognition tasks. An atlas guide/model-based segmentation approach is recognized as one of the most successful methods for image analysis [33–36]. So far, applications of an atlas based model include brain structures [37–41], soft tissue structures in the abdominal and pelvic area [32, 42], cardiac structures [29, 43, 44], and several bone structures [45–47], but not of a calcaneus. Shape representation is one of the most important issues in SSM based methods of medical image processing. Several shape representation forms are known [48]. Zhang and Liu [49] classify shape representation and description techniques into region-based methods and contour-based methods. Region-based methods consist of global methods such as geometric moment invariants, algebraic moment invariants, orthogonal moments, generic Fourier descriptors, grid based methods, and shape matrix as well as structural methods such as convex hull and medial axis. On the other hand, contour-based techniques exploit shape boundary including global methods such as simple shape descriptors (area, eccentricity, major axis orientation, blending energy), correspondence-based shape matching in space domain, shape signature (centroidal profile, complex coordinates, centroid distance, tangent angle, cumulative angle, curvature, area), boundary moments, elastic matching, stochastic method (autoregressive modeling), scale space method, and spectral transform as well as structural methods such as chain code representation, polygon decomposition, smooth curve decomposition, scale space method, syntactic analysis, and shape invariants. Shape representation forms are not limited to the classification proposed by Zhang and Liu [49]. Cottes et al. [25, 27, 28, 50] classified them as “Hand Crafted” models (built from specific scalable objects like lines, ellipses and arcs) [30], articulated models [51], active contour models [52], Fourier-based shape models [53], statistical models of shape, and final elements models [54, 55]. Also, for the 3D shape representation, Heiman and Meizer [56] considered medial models (pioneered by Pizer et al. [57]) that allow rendering 3D solids and spherical harmonics models [58–60].

The purpose of this work was to develop an anatomically accurate SSM of calcaneus which includes its morphological characteristics and provides mathematical representation of its shape.

## Methods

### Measurement setup

Retrospective data from regular hospital records have been used in this study. All patient records were anonymized and de-identified prior to processing according to the standard data release procedures of the two hospitals involved in the study. The Review Board of the Department of Radiology, Wroclaw Medical University, Wroclaw has approved the study. Poland Volume data have been acquired with three commercially available CT scanners (Siemens Somatom Definition Flash (2013), Forchheim, Germany; GE Medical Systems Discovery CT 750 HD (2010), Buckinghamshire, UK; and GE Medical Systems Light Speed CT VCT (2007), Buckinghamshire, UK). The tube voltage was 120 kVp in all three instruments. Data were retrospectively collected from the Department of Radiology at Regional Specialist Hospital, Research and Development Center and University Clinical Hospital in Wroclaw, Poland, saved in DICOM 3.0 format. All patients were scanned in feet-first, supine (FFS) position. Each volume image I, size [m, n, o] is made of different number of slices o, with each slice having size of 512×512 pixels. The parameters such as Pixel Spacing (0028, 0030)—xt, yt and Slice Thickness (0018, 0050)—zt were obtained from DICOM metadata [61] to extract physical dimensions of calcaneus. Volume data of 18 left and 18 right feet of 28 male subjects were collected. Subjects, aged from 17 to 62 years (mean 36.8 ± std 21.2 years), had no known foot pathology in the scanned foot. Subjects were aged matched, so they were no statistically significant differences between the age group averages of the left and the right foot subjects.

### Data processing

**Fig 1:**
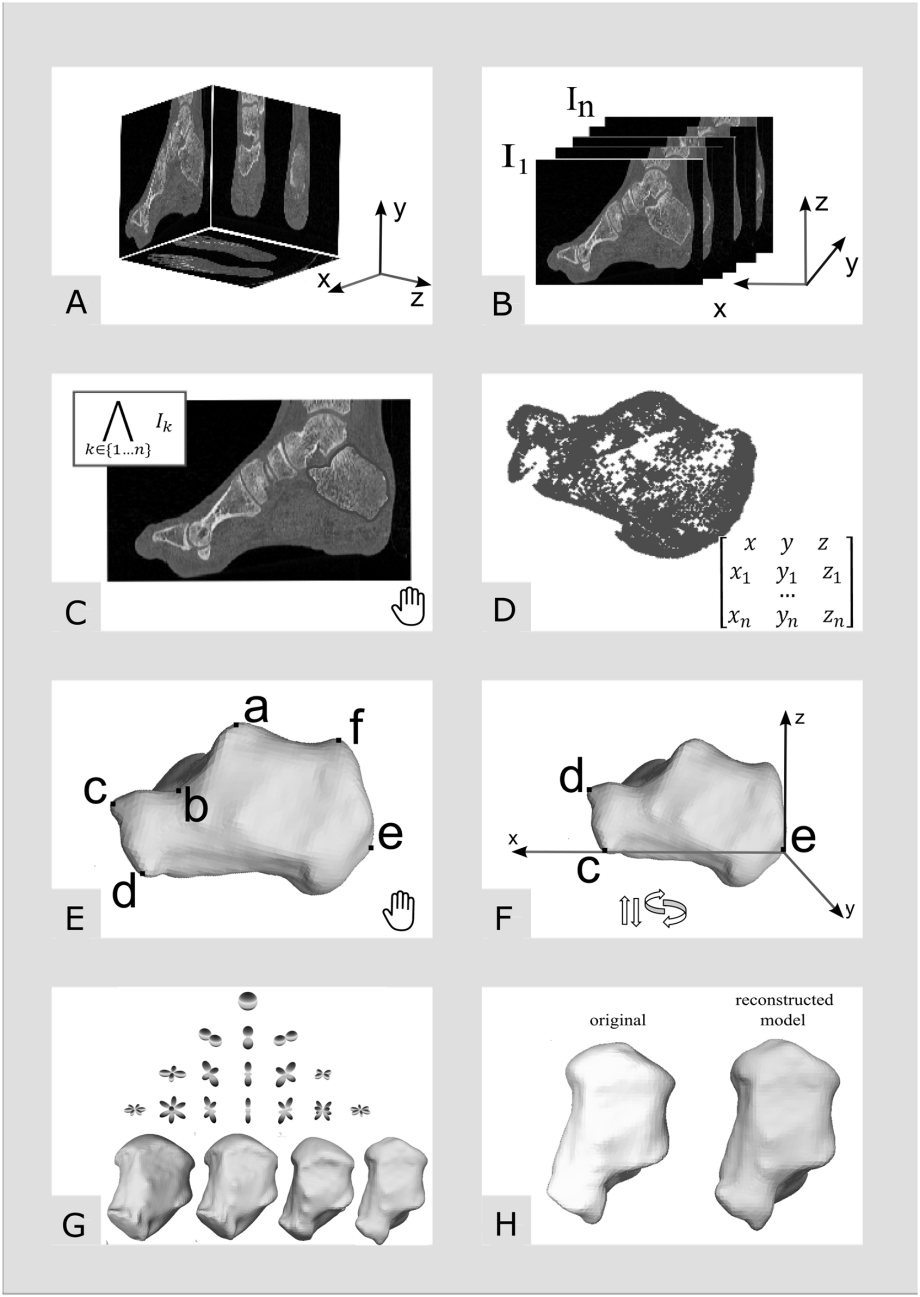
The process of building the morphometric and anatomical atlas of calcaneus. Hand icon denotes manual or semi-manual assisted processing lead by an experienced operator. Arrows icon denotes two isometric operations (translation and rotation) of the shape. Steps A to H are described in detail Section 2B.

A scheme for automatically building a morphometric and anatomical atlas was described by Subsol et al. [2]. It consists of the following stages: feature extraction, common feature identification, averaging feature position to obtain mean geometry and variability analysis. The process for building a morphometric and anatomical atlas of calcaneus is shown in Fig 1. In the following, we describe in detail the particular steps of the process. All procedures have been developed in Matlab (MathWorks, Inc., Natick, MA, USA) and are available from authors for free upon request.

1. Image pre-processing [Fig 1A and 1B] The volume image I is decomposed in the sagittal plane into a series of 2D images *I_k_*, *k* = 1, 2, …, *n*. Each *I*_*k*_ is first normalized in two steps:
  $$I_{k}-I_{kmin}\to I_{k},\frac{I_{k}}{I_{kmax}}\to I_{k}$$ (1)
  and then contrast is enhanced using sigmoid function [62]:
  $$S\left(I_{k}\right)=\frac{1}{1+\exp(g\left(c-I_{k}\right))}$$ (2)
  where *g* is a gain, and *c* is cut-off value, *I*_*kmax*_ is maximal and *I*_*kmin*_ is minimal pixel intensity value. Both parameters were set empirically to 0.4 and 0.7, respectively.
2. Contour extraction [Fig 1C]
  Extracting the contour of calcaneus in each image *I*_*k*_ can be achieved in a variety of ways. For the purpose of this study we use the region growing algorithm [63, 64] with manually assisted assignment of a seed point by an expert operator.
3. 3D point cloud to surface [Fig 1D]
  The result of the region growing algorithm is a set of coordinates *W* = [*x*, *y*, *z*], with *x* = [*x*1, *x*2, …, *xn*]′, *y* = [*y*1, *y*2, …, *yn*]′, and *z* = [*z*1, *z*2, …, *zn*]′ representing the contour of the calcaneus gathered respectively from each *I*_*k*_, *k* = 1, 2, …, *n*. For such a point-cloud set the oriented normals are estimated and then using Poisson surface reconstruction method the surface mesh is generated [65]. Meshlab (Pisa, Italy) software was used to generate meshes [66]. Surface representation is needed to further land-marking of the characteristic points of the calcaneus.
4. Land-marking [Fig 1E]
  Following Qiang et al. [7] the anatomic landmarks are manually marked on bone surface mesh (denoted by letters A to F in Fig 1E by an expert operator. They are: the highest point of the posterior articular facet (point A), the bottom of the posterior articular facet at the lateral surface intersecting the anterior process of the calcaneus (point B), the highest point of the calcaneocuboid joint (point C), the lowest point of the calcaneocuboid joint (point D), the most posterior point of the calcaneal tuberosity (point E), and the highest point on the superior edge of the calcaneal tuberosity (point F).
5. Averaging feature position and orientation [Fig 1F]
  The objects of study are not scanned in the same position so their orientation and location need to be normalized for the statistical bone atlas to be built. For this, the point-cloud set W and the characteristic points *C*, *D*, *E* are used. First, the plane *π*_1_ that includes points *C*, *D*, *E* is obtained
  $$\pi_{1}:\left(\vec{CE}\times \vec{DE}\right)\cdot \left(x-x_{C},y-y_{C},z-z_{C}\right).$$
  This is followed by calculating the angle *α* between the plane *π*_1_ and the plane *π*: *z* = 0. Then W is translated by the vector $\vec{E}=[x_{E},y_{E},z_{E}]$ to set the most posterior point of the calcaneal tuberosity (point *E*) in the origin. Next, *W* is rotated about the *x* axis by the angle *α*.
  The rotation matrix:
  $$r_{\alpha }=\left[\begin{array}{ccc} 1 & 0 & 0\\ \text{cos}\alpha & -\text{sin}\alpha & 0\\ 0 & \text{sin}\alpha & \text{cos}\alpha \end{array}\right]$$
  is applied and the angle *β* between *x* axis and vector $\vec{C}=[x_{C},y_{C},z_{C}]$ is calculated. Finally, *W* is rotated about the *x* axis by the angle *β* using the rotation matrix:
  $$r_{\beta }=\left[\begin{array}{ccc} \text{cos}\beta & -\text{sin}\beta & 0\\ \text{sin}\beta & \text{cos}\beta & 0\\ 0 & 0 & 1 \end{array}\right]$$
6. Spherical harmonics decomposition [Fig 1G] The mathematical shape description is achieved by using the spherical harmonics (SPHARM) [58, 67–70]. Using linear-in-parameters least square procedure, data (cloud of extracted points) are first fitted with a best sphere:
  $${\left(x_{d}-x_{O}\right)}^{2}+{\left(y_{d}-y_{O}\right)}^{2}+{\left(z_{d}-z_{O}\right)}^{2}-R_{BS}^{2}+\epsilon_{d}=0$$ (3)
  and then translated to a new coordinate system centred at the estimated origin $\left(\hat{x_{0}},\hat{y_{0}},\hat{z_{0}}\right)$:
  $$\left({\hat{x}}_{d},{\hat{y}}_{d},{\hat{z}}_{d}\right)\to \left(x_{d}-{\hat{x}}_{0},y_{d}-{\hat{y}}_{0},z_{d}-{\hat{z}}_{0}\right),d=1,2,...,D$$ (4)
  The center of the new origin is set as a center of the mass:
  $$\vec{r_{0}}=\frac{\sum_{k}\vec{r_{k}}m_{k}}{\sum_{k}m_{k}}$$ (5)
  Then, transformation from the Cartesian to spherical coordinates is made:
  $$\left({\hat{x}}_{d},{\hat{y}}_{d},{\hat{z}}_{d}\right)\to \left(\theta_{d},\phi_{d},R_{d}\right)$$ (6)
  $$\left({\hat{r}}_{d},{\hat{\theta }}_{d},{\hat{z}}_{d}\right)\to \left(\theta_{d},\phi_{d},R_{d}\right)$$ (7),
  where *θ*_*d*_ is the azimuth, *ϕ*_*d*_ is the elevation angle and *R*_*d*_ is the radius of sphere.
  Then the normalization to a unit sphere is performed:
  $$\left(\theta_{d},\phi_{d},R_{d}\right)\to \left(\theta_{d},\phi_{d},\frac{R_{d}}{R_{s}}\right)$$ (8),
  where *R*_*s*_ = *max*(*R*_*d*_)
  The complete complex-valued spherical harmonics are solutions to the Laplace equation:
  $$\Delta^{2}f=\frac{1}{r^{2}}\frac{\partial }{\partial r}r^{2}(\frac{\partial f}{\partial r})+\frac{1}{r^{2}\sin\theta }\frac{\partial }{\partial \theta }(\sin\theta \frac{\partial f}{\partial \theta })+\frac{1}{r^{2}\sin\theta }\frac{\partial }{\partial \theta }(\sin\theta \frac{\partial^{2}f}{\partial \theta^{2}})$$ (9)
  are given by:
  $$Y_{l}^{m}\left(\theta ,\phi \right)=N_{l}^{m}P_{l}^{m}\cos\left(\theta \right)e^{Im(\phi )},l\geq 0,\left|m\right|\leq l$$ (10)
  where $i=\sqrt{-1},N_{l}^{m}$ is the normalization factor defined as:
  $$N_{l}^{m}=\sqrt{\frac{(2l+1)(l-m)!}{4\pi (l+m)!}}$$ (11)
$P_{l}^{m}$ are the associated Legendre functions:
  $$P_{l}^{m}\left(x\right)=\frac{-1^{m}}{2^{l}l!}{\left(1-x^{2}\right)}^{m/2}\frac{d^{l+m}}{dx^{l+m}}{\left(x^{2}-1\right)}^{l}$$ (12)
  The real-valued spherical harmonics can be expressed as:
  $$y_{l}^{m}(\theta ,\phi )=\{\begin{array}{l} \sqrt{\left(2\right)}N_{l}^{m}P_{l}^{m}\text{cos}(\theta )\text{cos}(m\phi ),m>0\\ \sqrt{\left(2\right)}N_{l}^{m}P_{l}^{m}\text{cos}(\theta )\text{sin}(m\phi ),m<0\\ \sqrt{\left(2\right)}N_{l}^{0}P_{l}^{0}\text{cos}(\theta ),m=0 \end{array}$$ (13)
  A spherical harmonics based decomposition of the (*θ*_*d*_, *ϕ*_*d*_, *R*_*d*_/*R*_*s*_) calcaneus data can be expressed as:
  $$\frac{\hat{R_{d}}}{R_{s}}=\sum_{l=0}^{L}\sum_{m=-l}^{l}{\hat{c}}_{l}^{m}{\hat{y}}_{l}^{m}\left(\theta_{d},\phi_{d}\right),d=1,2,...,D.$$ (14)
  or alternatively:
  $$\frac{\hat{R_{d}}}{R_{s}}=\sum_{q=l}^{Q}{\hat{c}}_{q}y_{q}^{m}\left(\theta_{q},\phi_{q}\right),d=1,2,...,D.$$ (15)
  where *l* = ⌈*d*⌉ -1 and *m* alternates between *-l*, *l* + 1, …, 0, *l*-1, *l*.
  The coefficient estimates in the spherical harmonic expansion ${\hat{c}}_{q}$, *q* = 1, 2, … *Q* can be easily evaluated using the method of linear least-squares by concatenating the data into a single column vector as described earlier. The estimate of the spherical harmonics based calcaneus surface is then
  $${\hat{R}}_{d}\left(\theta_{d},\phi_{d}\right)={\hat{R}}_{dn}R_{s}$$ (16)
  Transforming this result back to Cartesian coordinate system:
  $$\left(\theta_{d},\phi_{d},R_{d}\right)\to \left({\hat{x}}_{d},{\hat{y}}_{d},{\hat{z}}_{d}\right)$$ (17)
  and then translating it back to the starting origin (see Fig 1G and 1H) creates an estimate of the calcaneus and allows to evaluate the quality of the approximation at the original points (*x_d_, y_d_, z_d_*), *d* = 1, 2, …, *D*.
7. Model and model order selection [Fig 1H] The optimal model order of the SPHARM expansion can be evaluated using one of the standard information criteria such as Akaike Information Criterion or the Rissanen Minimum Description Length (MDL) criterion [71, 72]. For the purpose of this works the latter criterion was used. Given the optimal model order, further reduction of the number of coefficients in the SPHARM expansion is achieved by applying a multiple hypotheses procedure to test each of the coefficient values for zero.

## Results

**Fig 2:**
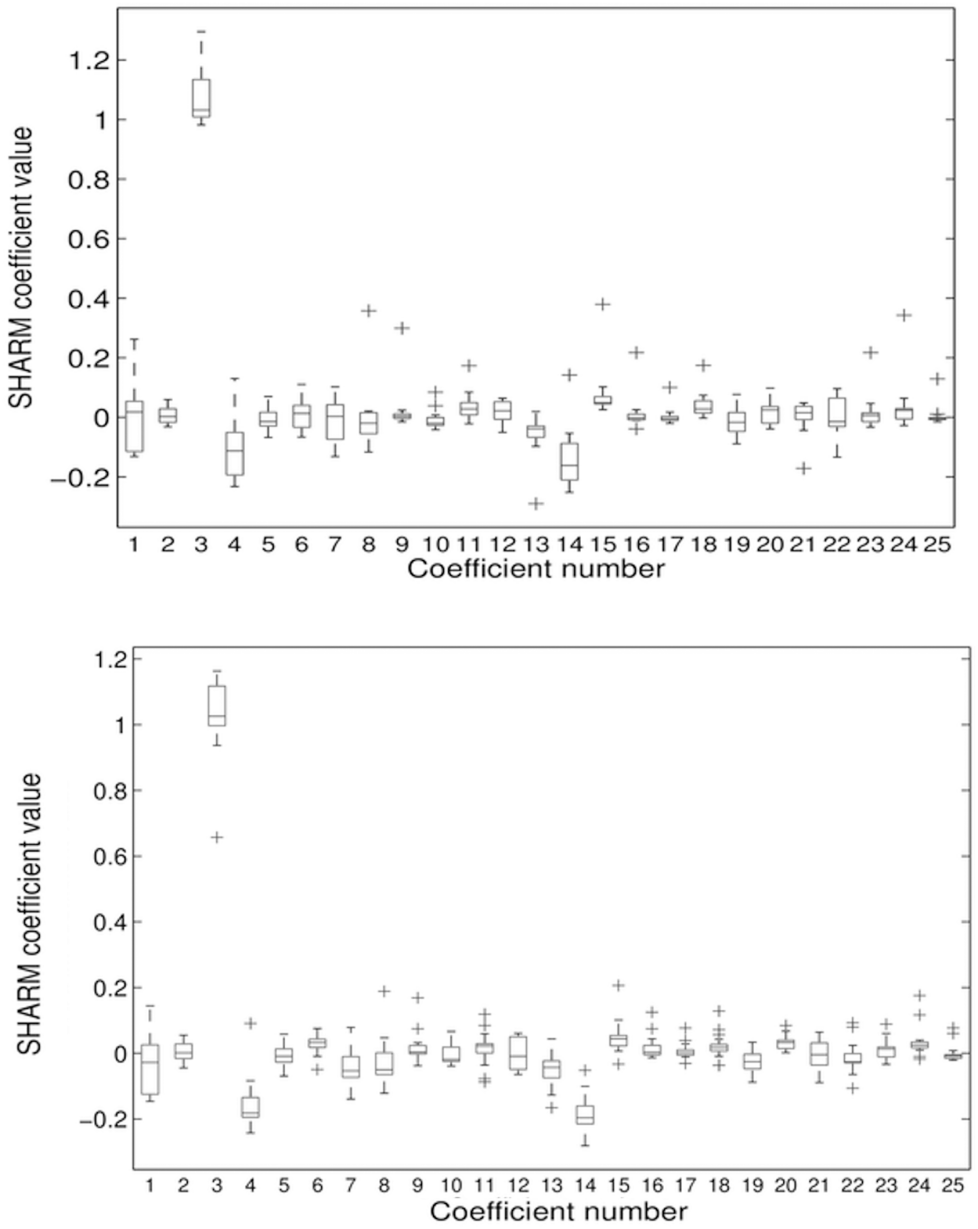
The statistics of SPHARM estimates (box-plots) of calcaneus (the first 25 coefficients) for the group of 18 left (top) and 18 right calcanei (bottom). Crosses indicate outliers.

**Fig 3:**
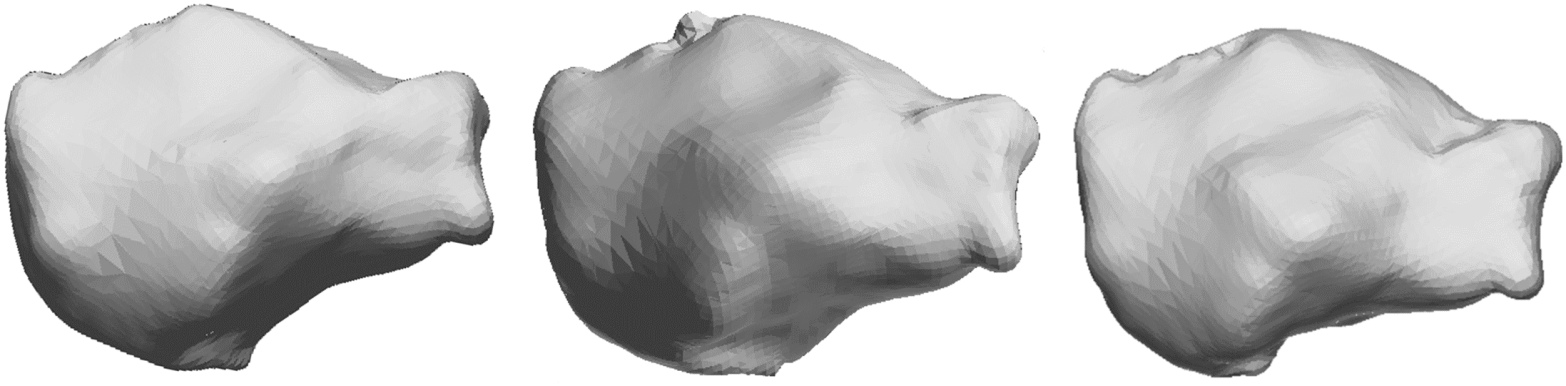
An example of reconstructed SSM of right calcaneus using (from left to right) the 25th, 50th and 75th quartile of the spherical harmonic coefficients.

**Fig 4:**
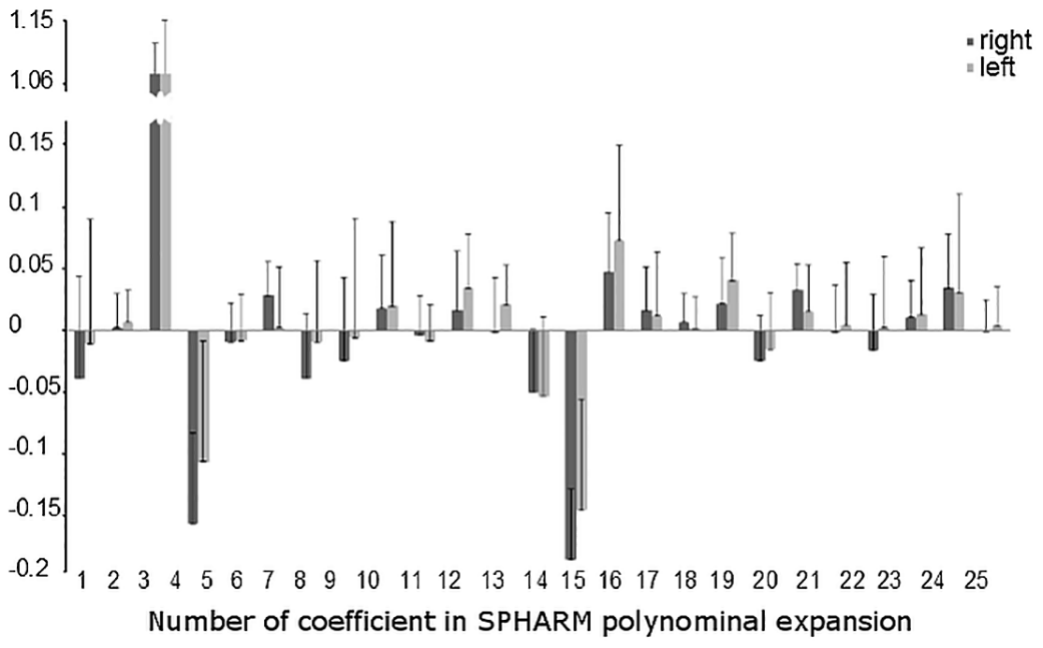
The mean values of SPHARM estimates of calcaneus (the first 25 coefficients) for the group of 18 right (dark gray) and 18 left (light grey) calcanei. Error bars indicate one standard deviation.

**Fig 5:**
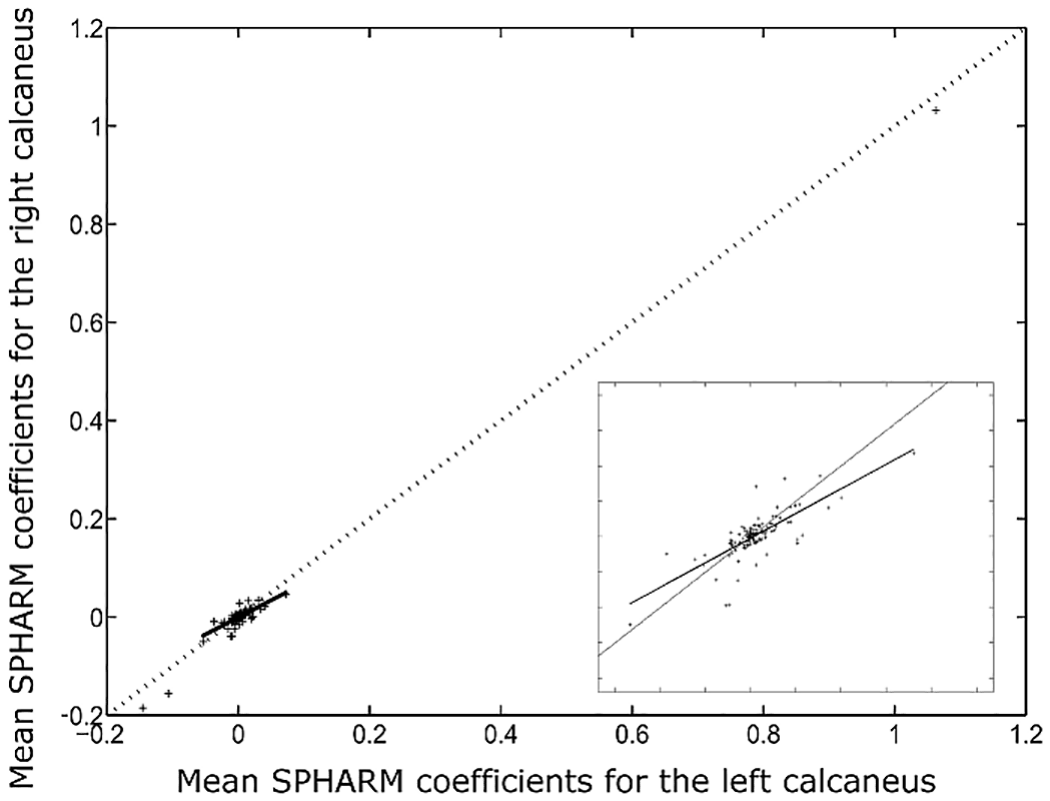
Correlation between the SPHARM coefficients of the left and the right calcaneus. Linear correlation is performed on the reduced set of coefficients excluding the three highest coefficients (see inset).

**Fig 6:**
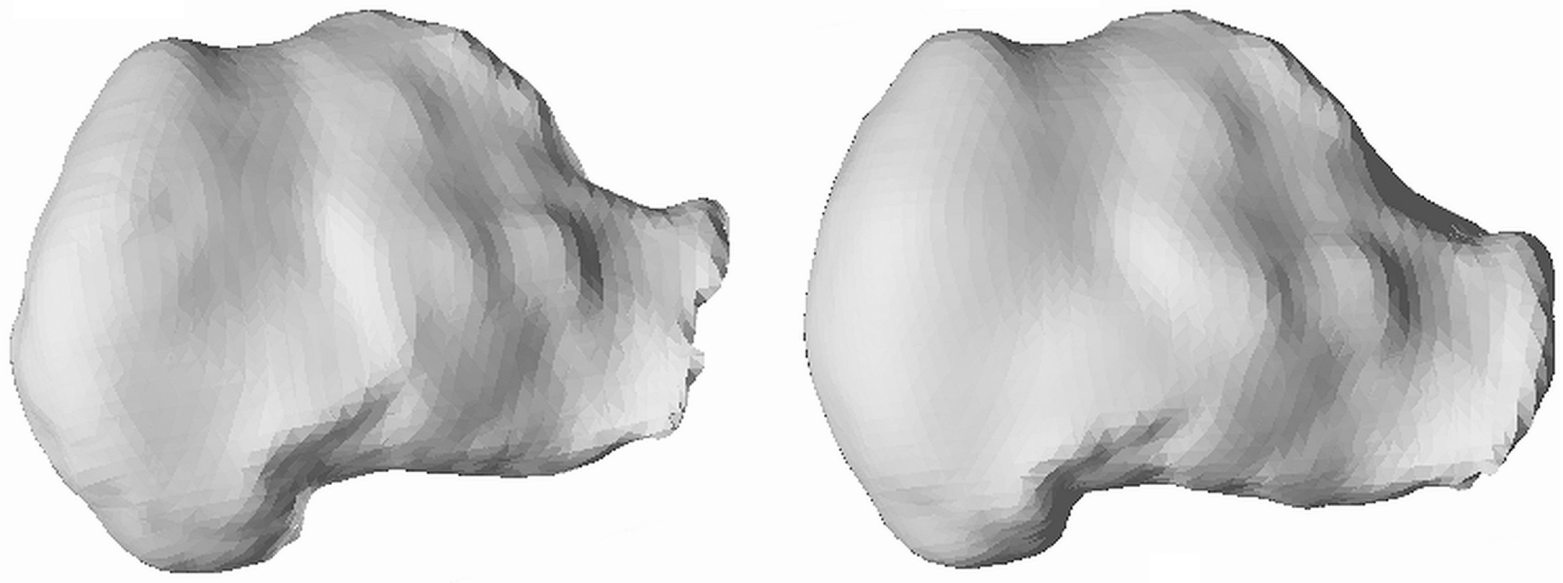
An example of reconstructed SSM of right calcaneus using the whole set of 144 spherical harmonic coefficients (left) and the reduced set of 41 representative coefficients (right).

The MDL criterion estimated the optimal radial order of SPHARM expansion at 11 resulting in 144 characteristic coefficients ${\hat{c}}_{l}^{m}$. Fig 2 shows the box-plots for the first 25 SPHARM coefficients (corresponding to radial order of 4) for the left and right bones. The first, second and third quartile of ${\hat{c}}_{l}^{m}$ were estimated and for them the model shape reconstructed. In Fig 3 we present reconstructed shape (as an example) for the right calcaneus. The mean values and the corresponding standard deviation (error bars) for the first 25 SPHARM coefficients are presented in Fig 4. Comparing the correspondent coefficients for the left and right bone we can see that some of them are similar and some of them have opposite sign. This is indicative of the symmetry of the objects. Fig 5 shows the correlation between mean values of SPHARM coefficients for left and right foot. When including the entire set of coefficients, the correlation of shapes is obviously very high (*r* ^2^ = 0.99) and statistically significant (*p* ≪ 0.01). This correlation remains moderate when the three highest coefficients omitted amounting to *r* ^2^ = 0.59, *p* ≪ 0.01 (see Fig 5). Application of a multiple hypotheses procedure to test each of the coefficient values for zero reduced the set of coefficient to 41 and 47 representative coefficients for the right and left calcaneus, respectively. Fig 6 shows an example of reconstructed SSM of right calcaneus using the whole set of 144 spherical harmonic coefficients and the reduced set of 41 representative coefficients.

Table 1 shows the morphological characteristics of the calcaneus based on landmarks distances measurement as follows (see Methods): the length of the calcaneal axis (LCA): the distance from point G (the midpoint between C and D) to point E, the height of the posterior facet (HPF): the perpendicular distance from point A to the calcaneal axis, the length of the posterior facet (LPF): the distance between A and B, the length of the anterior process (LAP): the distance between B and C, the height of the anterior process (HAP): the distance between C and D, the critical angle of Gissane (GA): ∠*ABC*, the tuber angle of Böhler (BA): (180°-∠*CAF*). No statistically significant differences were found between the parameters of the left and right foot (t-test, *p* > 0.05).

**Table 1:** The group statistics (MEAN±SD) of the morphological characteristics of the calcaneus based on landmark distances.

	both calcanei	left calcaneus	right calcaneus
LCA [mm]	80 ± 6	79 ± 7	81 ± 6
HPF [mm]	32 ± 5	33 ± 4	32 ± 6
LPF [mm]	27 ± 4	27 ± 4	28 ± 4
LAP [mm]	24 ± 3	23 ± 3	25 ± 3
HAP [mm]	24 ± 4	24 ± 3	25 ± 5
BA [deg]	37 ± 5	35 ± 5	39 ± 5
GA [deg]	120 ± 7	119 ± 6	120 ± 8

Abbreviations: the length of the calcaneal axis (LCA), the height of the posterior facet (HPF), the length of the posterior facet (LPF), the length of the anterior process (LAP), the height of the anterior process (HAP), the critical angle of Gissane (GA), the tuber angle of Böhler (BA).

## Discussion and Conclusions

Building an anatomically accurate statistical shape model of calcaneus requires close collaboration of image processing engineers, radiologists and orthopaedists. Following a general scheme for building such an atlas in an automatic function, as proposed by Subsol et al. [2], we were able to bring down the characterization of the mean geometry of calcaneus to several dozen representative coefficients of the spherical harmonic expansion. It should be noted that the scheme was not fully automatic and that the assistance of the experienced operator was necessary in two critical steps of building the model. That is, in the process of contour extraction where the selection of the seed point was manually assisted and in the difficult and tedious characterization of the representative anatomical features of the calcaneus. It is worth noting that the later was in a close agreement with the results of Qiang et al. [7] who also manually identified the anatomic landmarks of calcaneus. Shapes may be represented in many different ways and an argument can be made whether the choice of spherical harmonic representation was the most appropriate. Given the many advantages of spherical harmonics [69] such as their orthogonality and completeness, as well as the ability to estimate the coefficients using linear-in-parameters least-squares method provides a sufficient motivation for using this specific representation. It is important to acknowledge that other forms of 3D basis functions could also be used in place [73]. However, comparing different representations in terms of goodness-of-fit and the minimum number of representative coefficients, although interesting, was not within the scope of this work. One of the limitations of the study was the lack of complete CT data for both feet. It is expected that otherwise the symmetry of the model, as estimated via the two sets of spherical harmonic expansions, would be more evident. Another drawback was the acquisition of a single CT scanning per subjects (to limit the dose of radiation and cost), which prevented assessing the reproducibility of the measurement and modelling procedure. Nevertheless, other studies related to foot bone imaging with CT show relatively high test-retest reliability [24] that can be anticipated to hold for imaging calcaneus. Finally our study examined a set of male calcanei but the exact procedure can be readily applied for the female counterparts. To the best of our knowledge, the proposed statistical anatomical atlas of the calcaneus is presented for the first time. Tools of image processing, 3D shape modelling, and expert manual assistance were necessary to achieve the goal. The manual assistance in building a statistical shape model may appear to be a hindrance, but could be attempted, after verification, with robust shape registration methods [74, 75].

However, at the same time it provides an assurance of anatomical accuracy sought by medical professionals. In this view, the proposed statistical model of calcaneus including the quantitative mathematical shape description could be of value to diagnosing pathological changes by matching a model which contains information about the expected shape [76, 77]. It can be applied to monitoring growth process in childhood, bone and surrounding tissue age degeneration and osteoarthritis progression, as well as to detection of lesion (cysts, tumour-like changes). The model of calcaneus finds the application in pre-operative planning [78, 79], classifications [6] and follow-up of treatment of calcaneus fractures [8, 80], assessing the implant procedures [81, 82], and 3D reconstruction of bones [83]. Consequently, the model could also be used in image processing and computer vision software supporting those medical imaging tasks like segmentation or recognition.

As the summary demonstrates, there is already a considerable amount of 3D SSMs employed in medical image analysis. Extending this area to models of calcaneus could further support medicine in better diagnosis and treatment.

## Supporting Information

S1 Data Set The full data set for all 144 SPHARM coefficients (Fig 2). (XLS)

S2 Data Set Data used to reconstruction of 3D shape for the right calcaneus (Fig 3). (XLS)
